# Supplementary material for: Effect of timing of implementation of containment measures on Covid-19 epidemic. The case of the first wave in Italy
Source: PLoS One. 2021 Jan 29;16(1):e0245656. doi: 10.1371/journal.pone.0245656 (PMC7846280; doi:10.1371/journal.pone.0245656)
Supplement: Attachment — Submitted filename: Response to Reviewers.pdf [file pone.0245656.s001.pdf]

Editor

Plos One

December 24th, 2020

Dear Editor,

Thank you to give us the opportunity to resubmit our manuscript entitled “Effect of timing of implementation of containment measures on Covid-19 epidemic. The case of Italy” by L.Timelli and E. Girardi [PONE-D-20-34437].

The new submission follows the suggested PLOS ONE templates. Below are shown, point-by-point, the response (in bold) to your additional requirements. In a separate document please find a point-by-point letter to the reviewer’s comments.

It is of note that we are proposing a slight modification of the title given the current situation in Italy with the second epidemic wave. The new proposed title is “Effect of timing of implementation of containment measures on Covid-19 epidemic. The case of the first wave in Italy.”

1. Please ensure that your manuscript meets PLOS ONE's style requirements, including those for file naming.

Answer: we checked /changed the file naming according to PLOS ONE's style requirements

2. In your Methods section, please give the sources for the following information:
i) COVID-19 cases

Answer: we inserted the web link to the data as reported below:

https://github.com/pcm-dpc/COVID-19 (download 01/07/2020)

ii) ICU bed occupancy

Answer: we inserted the web link to the data as reported below:
https://www.trovanorme.salute.gov.it/norme/renderNormsanPdf?anno=2020&codLeg=74348&parte=1%20&serie=null

Furthermore, this is a retrospective study; thus, we ask that you revise the text (especially, but no limited to, the aims and Conclusions) to avoid unsupported statements.

Answer: text was revised to take into account of this comment

3. In your Data Availability statement, you have not specified where the minimal data set underlying the results described in your manuscript can be found; the link provided states data not found.

PLOS defines a study's minimal data set as the underlying data used to reach the conclusions drawn in the manuscript and any additional data required to replicate the reported study findings in their entirety. All PLOS journals require that the minimal data set be made fully available. For more information about our data policy, please see http://journals.plos.org/plosone/s/data-availability.

Upon re-submitting your revised manuscript, please upload your study's minimal underlying data set as either Supporting Information files or to a stable, public repository and include the relevant URLs, DOIs, or accession numbers within your revised cover letter. For a list of acceptable repositories, please see http://journals.plos.org/plosone/s/data-availability#loc-recommended-repositories. Any potentially identifying patient information must be fully anonymized.

Important: If there are ethical or legal restrictions to sharing your data publicly, please explain these restrictions in detail. Please see our guidelines for more information on what we consider unacceptable restrictions to publicly sharing data: http://journals.plos.org/plosone/s/data-availability#loc-unacceptable-data-access-restrictions. Note that it is not acceptable for the authors to be the sole named individuals responsible for ensuring data access.

We will update your Data Availability statement to reflect the information you provide in your cover letter.

Answer: as reported above, used data are available at the links provided:

Covid-19 cases → https://github.com/pcm-dpc/COVID-19 (downloaded 01/07/2020)

ICU bed → https://www.trovanorme.salute.gov.it/norme/renderNormsanPdf?anno=2020&codLeg=74348&parte=1%20&serie=null (Table 1)

Regional population at 01/01/2020 → www.demo.istat.it

4. Please upload a new copy of Figure 2 as the detail is not clear.

Answer: The new figure 2 is now provided with high pixel resolution

5. Please include your tables as part of your main manuscript and remove the individual files. Please note that supplementary tables should be uploaded as separate "supporting information" files.

Answer: we included the tables as part of the manuscript.

We hope that the manuscript meets with your and the referees' approval and it is suitable for publication in Plos One.

Looking forward to hearing from you.

Sincerely yours,

Dr. Laura Timelli,

Department of Epidemiology

IRCCS "L. Spallanzani"

Via Portuense, 292 – 00149– Rome, Italy

E-mail: laura.timelli@inmi.it

Phone: +39-06-55170924

Point-by-Point letter to the reviewer's comments ("Effect of timing of implementation of containment measures on Covid-19 epidemic. The case of Italy" by L.Timelli and E. Girardi [PONE-D-20-34437])

Reviewer #1: The authors present a study that shows the impact of the timing of implementation of non-pharmaceutical containment measures (NPMs) on Covid-19 epidemic. They focus on the italian national lockdown, which started on March 11th 2020 and ended on May 4th 2020. The impact of NPMs on healthcare system has been evaluated in 21 italian local administrations (19 regions+2 autonomous provinces). Three indices have been used in order to assess this impact: cumulative incidence (CI), intensive care units occupancy (percentage on the total amount of ICUs), mortality rate. Timing of implementation of NPMs has been calculated as the number of days between the date on which each region reached a CI equal to 1.0 per 100k inhabitants, and the date of National Lockdown (it has been called delay). In the following lines some comments about the manuscript are reported:

1) The impact of the delay on healthcare system in terms of ICU occupancy and mortality rates is not sufficiently assessed. Two scatterplots (as Fig. 3) should be added: one between delay and highest ICU occupancy, the other one between delay and mortality rates (at May 11th). Related correlations should also be reported.

Answer: we followed your suggestion and added two scatterplots, one between delay and highest ICU occupancy, the other one between delay and mortality rates (at May 11th) and relative correlations. Following also your comment number 2, we added the scatterplot between delay and the number of ICU beds occupied per 100k inhabitants (hereinafter referred to as maximum ICU beds occupancy rate) and relative correlation. We included in the revised version another figure (Figure 5) combining these new scatterplots in three panels. Also Table 1 was updated adding a column with maximum ICU beds occupancy rate. We also revised methods, results and discussion sections to take into account the new results.

2) Looking at the data on Table 2, correlation between delay and mortality rate is about 0.7, which is close to the correlation between delay and CI. On the other hand, correlation between delay and highest percentage of ICU occupancy is lower (0.38). This probably means that the percentage of ICU occupancy is not the right statistic, and it should be replaced by the number of ICU beds occupied per 100k inhabitants, if these data are available. Percentage in fact is heavily affected by the total amount ICU beds, which is more closely linked to the quality level of the healthcare system than the spread of the epidemic.

Answer: Thanks for the advice. We calculated also the maximum ICU bed occupancy rate and relative correlation. We decided to maintain also the previous statistic as a measure both of quality level of the healthcare system and the impact of the spread on HS. It is of note that the correlation between the highest percentage of ICU occupancy maximum ICU bed occupancy rate is high (0.95). Below we show the scatterplot. We also revised methods, results and discussion sections to take into account the new results.

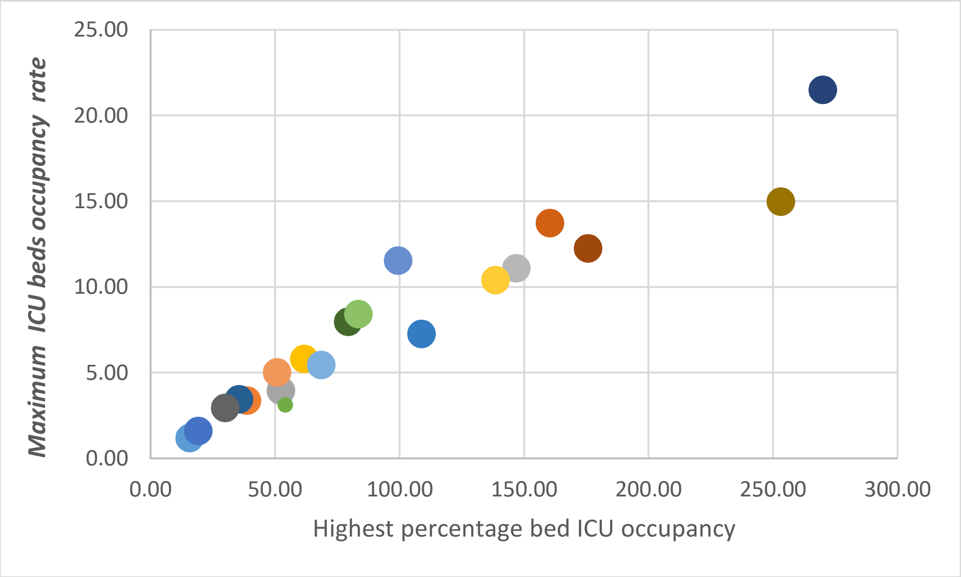

3) NPMs effectiveness can be seen after 10 – 31 days (Fig. 1, 2 and 4), regardless of the delay in their implementation. The authors should put a little bit more emphasis on this result, in the abstract and in the Results section.

Answer: thanks for the suggestion. This is now highlighted in the revised version.

4) Figure 2 has a very low pixel resolution, it is almost unreadable. Moreover, daily cases have too many fluctuations: it would be better to replace them with weekly averages or moving averages.

Answer: The new figure 2 is now with high pixel resolution; we also used weekly moving average, as suggested.

5) References are ok.

Answer: Thanks for the comment.

Reviewer #2: This interesting paper explores how the timing of implementation of NPMs (or NPIs) impacted the cumulative incidence, ICU bed occupancy and mortality related to COVID-19 in Italy. I found the work clear and sufficiently explicative, providing support to modelling studies that suggested that the timing of implementation of control measures is critical to achieving effective control of the epidemic.
Among the strengths, I would mention the clarity of the aims and among the limitations, I would certainly agree that the quality of the data, aggregated, did not allow for in-depth analysis such as age and sex standardization.

The manuscript is very linear and coherent. Moreover, it is technically sound, and the data support the conclusions. Moreover, general conclusions are acceptable and in line with recent literature reports. I particularly appreciated the mention of the data coming from the Italian sero-survey and

the report on excess mortality in the limitations section, as it gives a realistic picture of the reality of the pandemic in Italy while acknowledging the struggle of gathering complete data on new cases and deaths.
I believe the statistical analysis has been performed appropriately and rigorously. However, I would add a few details in the Methods/statistical analysis section, both technical, such as the retrospective nature of the study, and on aspects such as the specific software used to perform the analysis

Answer: we thank the reviewer for these positive comments; following your suggestions, we specified that the study is retrospective and reported the software used for the analysis.

In Table 1 I would better explain the case-based measures, which do not seem clear.

Answer: we better specified the case-based measures, as suggested.

The authors made all data underlying the findings in their manuscript fully available.
The manuscript is presented in an intelligible fashion and written in standard English, however, I would proofread it as a few sentences might be less clear if some typos are not corrected (e.g. figure 3 "shows a scatterplot by Region of the relationship of CI two months later the date of lockdown", I believe it should be 2 months "after"), as well as some past tense forms or the s for the third person form of some verbs.

Answer: the new version was further revised by a mother tongue person aiming to improve the language and to correct the typos.

The aims, the discussion and the conclusions are coherent and sound, and ultimately support the adoption of early containment measures in order to counteract the epidemic surge and control its magnitude.

Answer: thanks for the positive comment.
